# The influence of claw morphology on gripping efficiency

**DOI:** 10.1242/bio.059874

**Published:** 2023-05-16

**Authors:** Graham Turnbull, Sutejas Chari, Zehao Li, Ziyue Yang, Catharina Maria Alam, Christofer J. Clemente, Parvez Alam

**Affiliations:** ^1^ School of Engineering, The University of Edinburgh, Sanderson Building, Robert Stevenson Road, The King’s Buildings, Edinburgh EH9 3FB, UK; ^2^ School of Applied Sciences, Edinburgh Napier University, 9 Sighthill Ct, Edinburgh EH11 4BN, UK; ^3^ School of Science, Technology and Engineering, University of the Sunshine Coast, Sippy Downs, 4558, QLD, Australia

**Keywords:** Lizard claw, Morphology, Climbing

## Abstract

This paper considers the effects of claw morphology on the gripping efficiency of arboreal (*Varanus varius*) and burrowing (*Varanus gouldii* and *Varanus panoptes*) lizards. To ensure a purely morphological comparison between the lizards, we circumvent the material effects of claws from different species, by modelling and testing claw replicates of the same material properties. We correlate climbing efficiency to critical morphological features including; claw height (*h*_*c*_), width (*w*_*c*_), length (*l*_*c*_), curvature (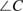) and tip angle (*γ*), which are expressed as ratios to normalise mechanically beneficial claw structures. We find that there is strong correlation between the static grip force *F*_*sg*_ and the claw aspect 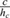 and the cross-sectional rigidity ratio 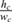, and milder correlation (i.e. higher scatter) with the profile rigidity ratio 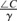. These correlations are also true for the interlocking grip force *F*_*int*_ over different shaped and sized protuberances, though we note that certain protuberance size-shape couplings are of detriment to the repeatability of *F*_*int*_. Of the three lizard species, the claws of the arboreal (*V. varius*) are found to be superior to those of the burrower lizards (*V. gouldii* and *V. panoptes*) as a result of the *V. varius* claws having a smaller aspect, a higher cross-sectional rigidity ratio and a small profile rigidity ratio, which are deemed noteworthy morphological parameters that influence a claw's ability to grip effectively.

## Teaser

**Summary:** By 3D printing claw shapes, we decouple the effects of geometry and materials. Through experimental testing, we show that certain claw morphologies improve grip strength more effectively than others.

## INTRODUCTION

Climbing animals in the natural world exhibit diverse gripping mechanisms (Dai et al., 2002; Heethoff and Koerner, 2007; Lautenschlager, 2014), each of which has likely evolved to cater for differences in size, weight and the environments in which they climb. While some lizards like geckos use van der Waals mechanisms to improve adhesive grip strength on smooth substrates, the use of claws as mechanical grips on rough substrates might be a more effective strategy for weightier animals such as monitor lizards (Guo et al., 2012). Research efforts on animal claws have to date, highlighted the relationships between claw morphology, habitat and lifestyle (Pike and Maitland, 2004; Stork, 1983; Zani, 2000; Cartmill, 2013; Birn-Jeffrey et al., 2012). Climbing birds for example, have been found to have more distinctly curved talons than ground-dwelling birds (Pike and Maitland, 2004), while arboreal anoles are reported to have longer claws and a larger claw base-height when compared to claws from non-arboreal anoles (Crandell et al., 2014). Zani (2000) correlated claw morphology and clinging performance and found that increases in claw curvature and toe width improved clinging performance to smooth surfaces, while increases in claw height and decreases in toe length improved clinging performance on rough substrates. Claw arc and tip angle are considered critical parameters for mechanical strength in bird talons (Tinius and Patrick Russell, 2017) and are likely important parameters in lizard claws as well (Glen and Bennett, 2007; Pike and Maitland, 2004), along with claw height and curvature (Crandell et al., 2014), which have been found to correlate with tree diameter and height, respectively (Yuan et al., 2019). The findings of Yuan et al. (2019) align closely to the morphometric work of D'Amore et al. (2018), who posited an intimate relationship between claw geometry and the lizard's habitat. In insects, it has been shown that surface roughness (Dai et al., 2002; Ditsche-Kuru et al., 2012) influences claw grip strength, with parameters such as claw tip diameter and tip sharpness identified as key morphological parameters in gripping (Pattrick et al., 2018). These works further consolidate the general hypothesis that claws have adapted to meet specific functional requirements relative to the environment.

Differences in claw morphologies can change claw-substrate interactions during climbing, and thus mechanical gripping efficiency. Arboreal species typically have longer, more curved and pointed claws to penetrate and hold onto exposed leading surface edges. This allows them to create a non-vertical contact (gripping) surface perpendicular to the adductor force, supporting a pull to enable climbing (D'Amore et al., 2018). As such, penetration and protuberance grip are valuable parameters to consider when evaluating the gripping performance of claws on specific substrates, and the consequent benefits to climbing. Of course, not all climbing occurs on penetrable surfaces. Rather than penetrating a rock surface, rock-field lizards for example, use their claws to interlock with sandstone or rock particles when climbing. Worn down claws are rarely observed in rock-dwelling taxa (D'Amore et al., 2018), indicating that claw-substrate sliding occurs infrequently, if at all.

**Fig. 1.**
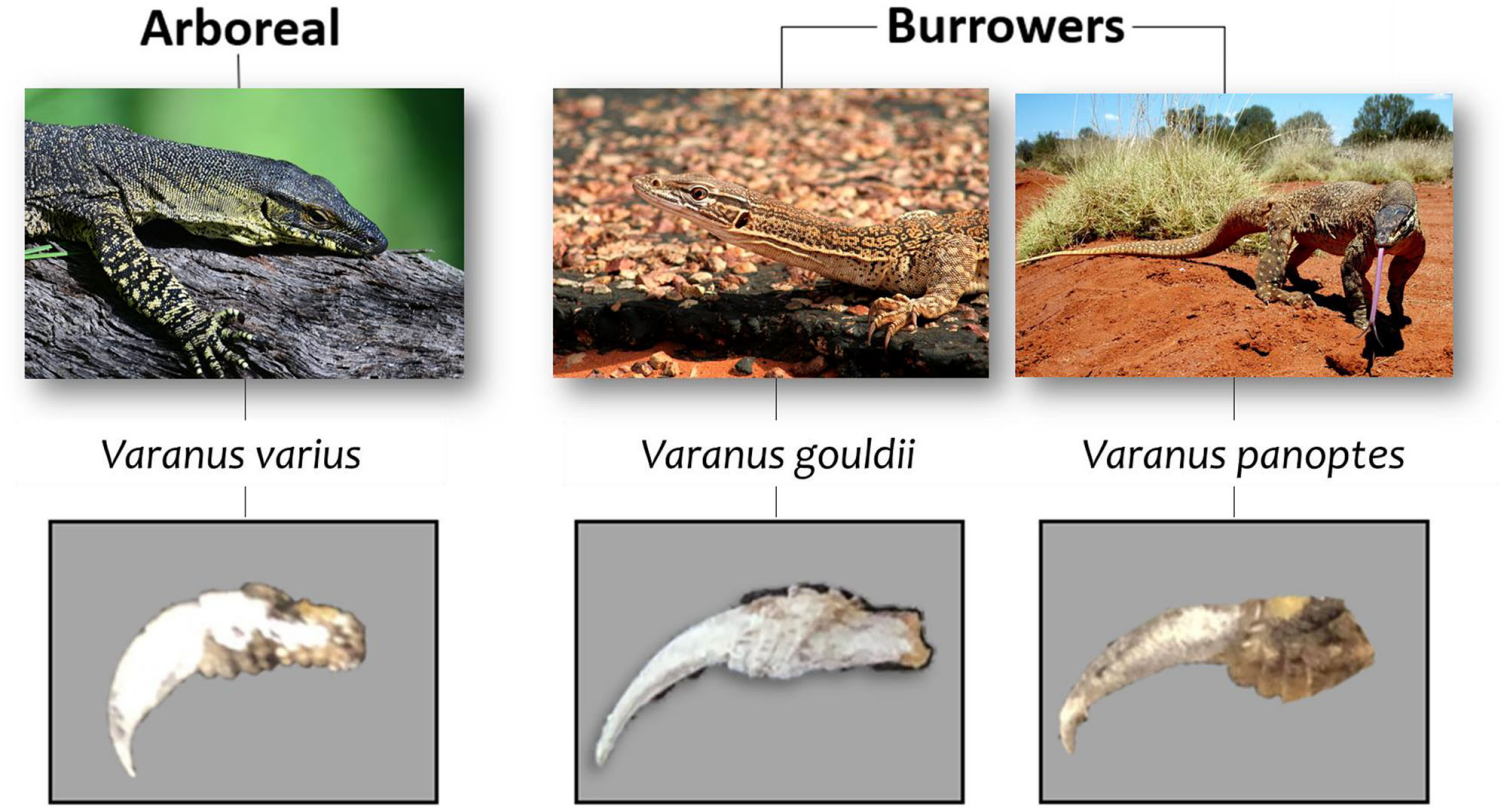
Pictures of *V*. *varius*, *V*. *gouldii* and *V*. *panoptes*, and their claws.

Though Zani (2000), as well as other researchers such as Tulli et al. (2011) and Crandell et al. (2014), reported a positive correlation between claw base-height and a lizards' maximum clinging force, their tests were conducted on live lizards. While their work revealed important information, testing on live lizards fails to isolate the specific role of claws since there are many independent variables including muscle strength, temperament, levels of fatigue, age and sex. As such, it is difficult to accurately deduce how the different morphological parameters of a claw influence its gripping performance. In light of this, we aim in this paper to isolate claw interaction with a substrate as a means to yielding more specific interpretations on how claw morphology might influence gripping. Our research will focus solely on the claw morphologies in relation to gripping in Varanids (i.e. monitor lizards in the genus Varanus). To do this, we will need to eliminate differences arising as a result of materials properties. These lizards have successfully adapted to Australia's diverse ecosystems and are now the top predator in many regions (King and Green, 1999). One essential reason for the Varanids' success may be their use of highly adapted claws for climbing and burrowing in a variety of environments. In this paper, we consider claws belonging to three different species, *Varanus varius* (arboreal), *Varanus gouldii* (burrower) and *Varanus panoptes* (burrower), Fig. 1. While the claws of burrowers are adapted for sprinting and extensive digging, arboreal species have claws that may be better for puncturing wood (Clemente et al., 2012; Thompson et al., 2009). This is because while burrower species claws typically display a gentle curvature, claws from arboreal species tend to be more tightly curved with a prominently pointed tip. As such, it can be said that microhabitat is related to claw morphology as evidenced by Tulli et al. (2009) A few details on each species in our research follows.

### *Varanus varius*, an arboreal Varanid

*V. varius* is found in both open and closed forests, can forage over distances of up to 3 km a day, and is adept at climbing in trees where it hunts its prey (Cogger, 2014; Guarino, 2002). This Varanid is found in eastern Australian forests and woodlands from Queensland in the north to Gippsland in the south, and from western New South Wales to Broken Hill (Cogger, 1992). They are the second largest Australian monitor lizard, growing up to 2 m in length and weighing up to 14 kg. *V. varius* is adept at climbing and may have adapted claws to compensate for its size and weight (Labonte and Federle, 2015).

### *Varanus gouldii*, a burrowing Varanid

*V. gouldii* is found ubiquitously across Australia, but predominantly in the northern and eastern regions where they inhabit grasslands and woodlands. This species spends much of its time in sandy soils and is limited in its ability to climb trees. *V. gouldii* typically grows to 140 cm and can weigh up to 6 kg (Pianka and King, 2004). The large geographical spread of this species may relate to its migratory behaviour, which can be attributed to the exhaustion of resources (Limpus, 1995; Thompson, 1992, 1995). Using its claws to forage in dense leafy detritus, this Varanid will retreat to an underground burrow if either ambient or soil temperatures are too high.

### *Varanus panoptes,* a burrowing Varanid

*V. panoptes* is preponderant in the areas of northern Australia, western Australia and Queensland. While *V. gouldii* is typically restricted to sandy soils, *V. panoptes* resides comfortably in a range of different habitats. The size of *V. panoptes* varies greatly between the sexes, with females and males growing to an average length of 90 cm and 140 cm, respectively (Pianka and King, 2004), and can weigh up to 7 kg (Auliya and Koch, 2020). This Varanid will typically shelter in self-dug burrows, but it has also been observed resting in hollow trees. While it is predominantly terrestrial, it can occasionally be observed climbing trees, though with less agility and at lower speeds than arboreal Varanids. When *V. panoptes* does climb, it tends to do so on milkwood trees, which have a rather soft (corky) bark (Pianka and King, 2004). Similar to *V. gouldii*, *V. panoptes* digs with its forelimbs, using its hindlimbs to push away loose substrate (Pianka and King, 2004).

## RESULTS

**Fig. 2.**
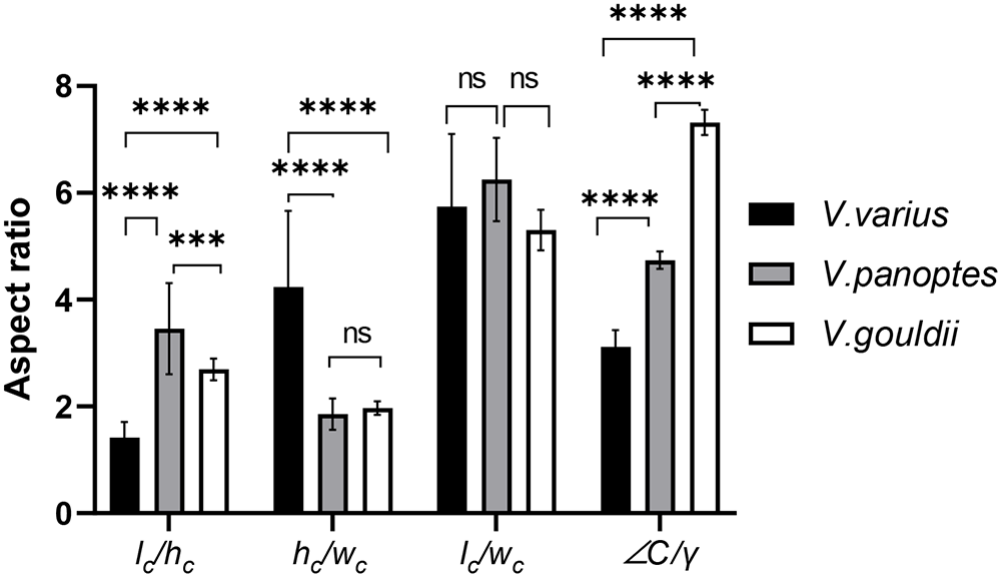
**Claw aspect ratio (**
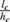
**), cross-sectional rigidity ratio (**
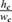
**), lateral stability (**
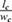
**), and the profile rigidity ratio (**
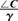
**) for *V. varius* (black bars, *n*=27), *V. gouldii* (grey bars, *n*=14) and *V. panoptes* (white bars, *n*=10).** The bars indicate mean values ±s.d. Asterisks (*) denote statistically significant differences between the mean values of specified groups. ****=*P*<0.0001, ***=*P*<0.001, ns=not significant as calculated using one-way ANOVA and Tukey's post hoc test.

Table 1 comprises the key measurements of the original claw samples for each of the three species and includes both standard deviations (s.d.) and coefficients of variation (CoV) for each sample set. *V. varius* stands out as it has by far the largest tip angle, while the claw length of *V. panoptes* is 1.8-fold longer that of *V. gouldii* and 2.6-fold longer than that of *V. varius*. *V. panoptes* also exhibits the greatest mean claw height, being marginally greater than that of *V. varius* and each is within one standard deviation from the arithmetic mean of the other. In addition, it can be noted that the claw curvature is smaller in *V. varius* than it is for either of the burrowing lizard species. Ratios of the mean values of different morphological measurements from Table 1 are presented in Table 2 and consist of 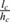, 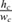, 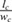, and 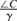 for *V. varius* (*n*=27), *V. gouldii* (*n*=14), *V. panoptes* (*n*=10). These same ratios are also applied to the 3D printed claws to ascertain the closeness of the 3D printed claws to the real ones, and we note that the 3D printed claws exhibit only between 1-8% difference from the ratios calculated for the real claws. The arboreal lizard *V. varius*, exhibits a significantly lower (*P*<0.0001) 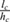 than the other two species indicating that the claws of these lizards have a lower aspect (i.e. are stubbier) than those of the burrowing species *V. gouldii* and *V. panoptes*, the latter of which has the highest aspect cf. Fig. 2. The ratio 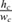, is a cross-section indication of claw rigidity (hereinafter: cross-sectional rigidity ratio), and we note in Table 2 and Fig. 2 that this ratio is significantly higher (>2-fold, *P*<0.0001) in the claws of the arboreal lizard *V. varius* than it is in either of the burrowing species. The 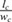 ratio is indicative of lateral stability and here, we note that *V. panoptes* has the highest 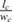, followed by *V. varius* and *V. gouldii*, respectively. However, these differences are not statistically significant as is evident in Fig. 2. Finally, 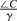 is a profile-view indication of claw rigidity (hereinafter: profile rigidity ratio). This is because (a) structures with greater curvatures have to carry bending moments, shear forces and torsional moments, and as the curvature is decreased the torsional moment reduces until it reaches a zero-value (for flat structures where there is only shear and bending), and (b) a larger tip angle means there is more material at the tip in the direction of claw loading.

**Table 1.**
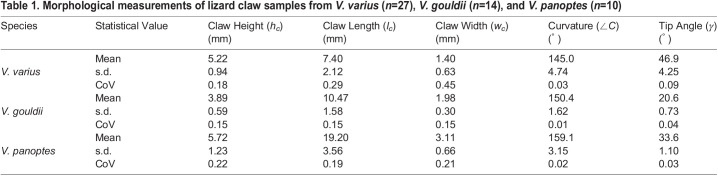
Morphological measurements of lizard claw samples from *V. varius* (*n*=27), *V. gouldii* (*n*=14), and *V. panoptes* (*n*=10)

**Table 2.**
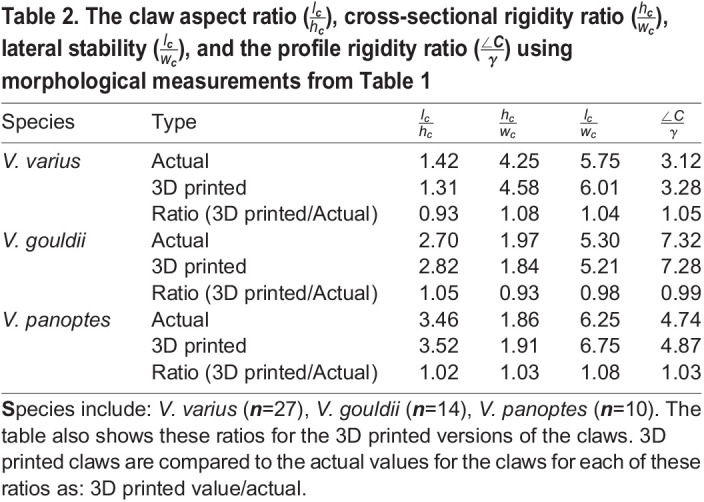
**The claw aspect ratio (** 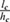 **), cross-sectional rigidity ratio (** 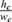 **), lateral stability (** 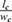 **), and the profile rigidity ratio (** 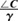 **) using morphological measurements from Table 1**

**Fig. 3.**
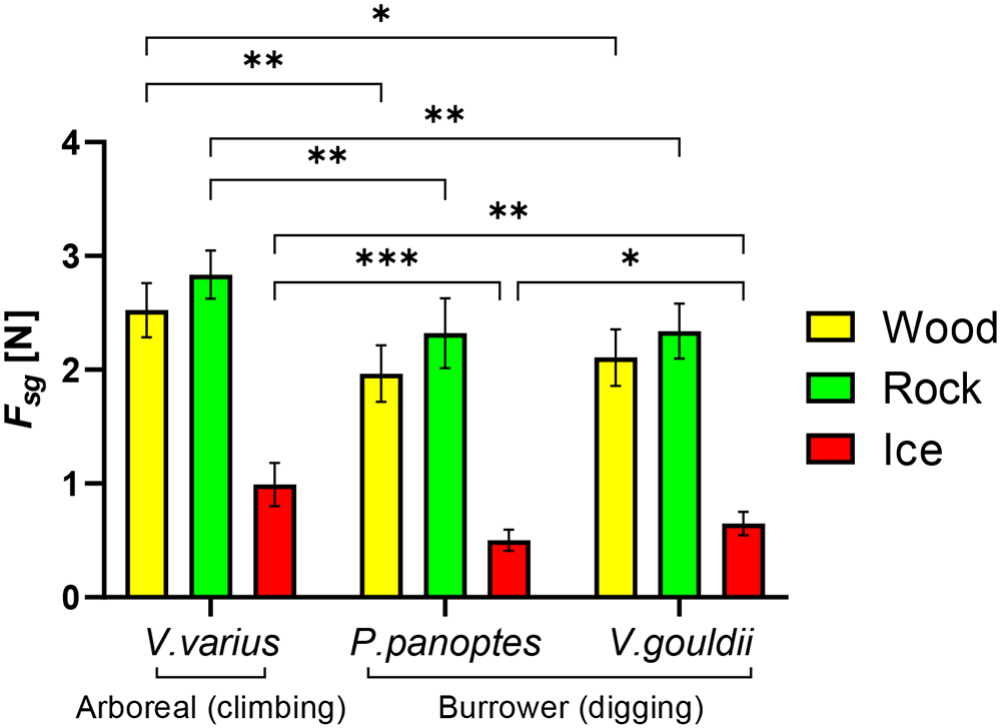
**Static grip measured for each claw (*n*=8 for each species) on different substrates.** The bars indicate mean values ±s.d. Asterisks (*) denote statistically significant differences between mean values of specified groups. ***= *P*<0.001, **= *P*<0.01, *= *P*<0.05, calculated using two-way ANOVA and Tukey's post hoc test.

**Fig. 4.**
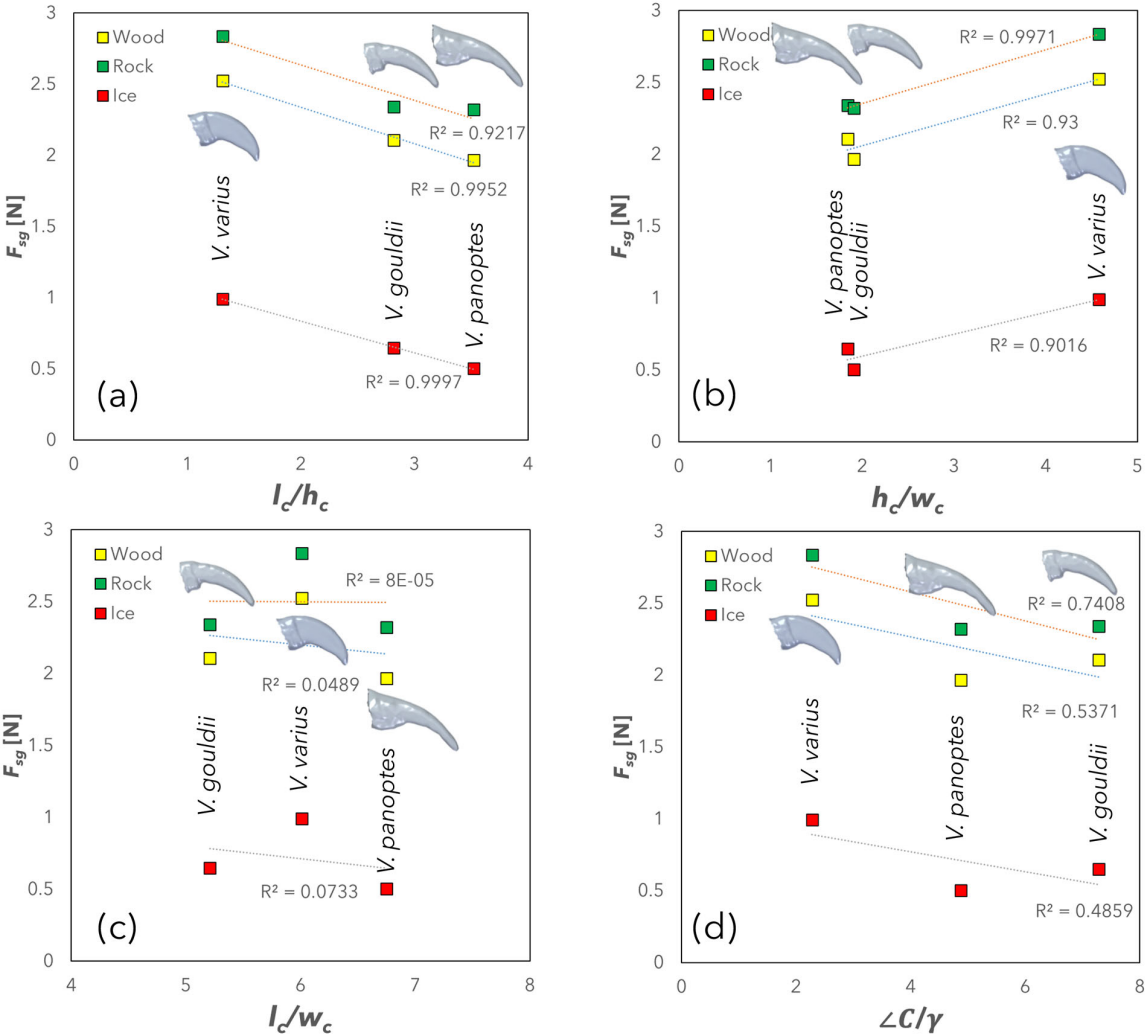
**Mean static grip (*n*=8 for each point) plotted against** 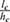
**,** 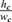
**,** 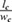
**, and** 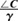 **(cf. Table 2) for each of the lizard species.**

A histogram, Fig. 3, provides details on the static grip (*F*_*gs*_) for each of the claw types on wood, rock and ice substrates. A higher average force is indicative of superior gripping performance, since it represents a claw's ability to support load prior to slipping. *V. varius* outperforms the burrower species across all three surfaces, while no significant difference was observed between *V. panoptes* and *V. gouldii*, except on the ice surface, where *V. panoptes* performed worse than *V. gouldii* (*P*<0.05). *V. varius* exhibits static grip 21.6%, 21.3%, and 52.3% higher in wood, rock, and ice substrates, respectively, than the next best performing claw (*V. gouldii*). Static grip forces were significantly lower for all claw types on the ice surface but were not significantly different between rock and wood. These are plotted in Fig. 4 against (a) 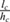, (b) 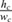, (c) 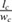, and (d) 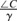 (*n*=8 for each point). In this figure, we note that while the static grip force is inversely correlated to aspect ratio (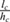) and positively correlated with the cross-sectional rigidity ratio (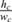) with low levels of scatter (as noted by the high determination coefficients), there is no observable correlation between static grip force and lateral stability (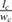), and the negative correlation between static grip force and the profile rigidity ratio (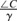) exhibits a relatively high scatter (as is noticeable from the observably low coefficients of determination).

**Fig. 5.**
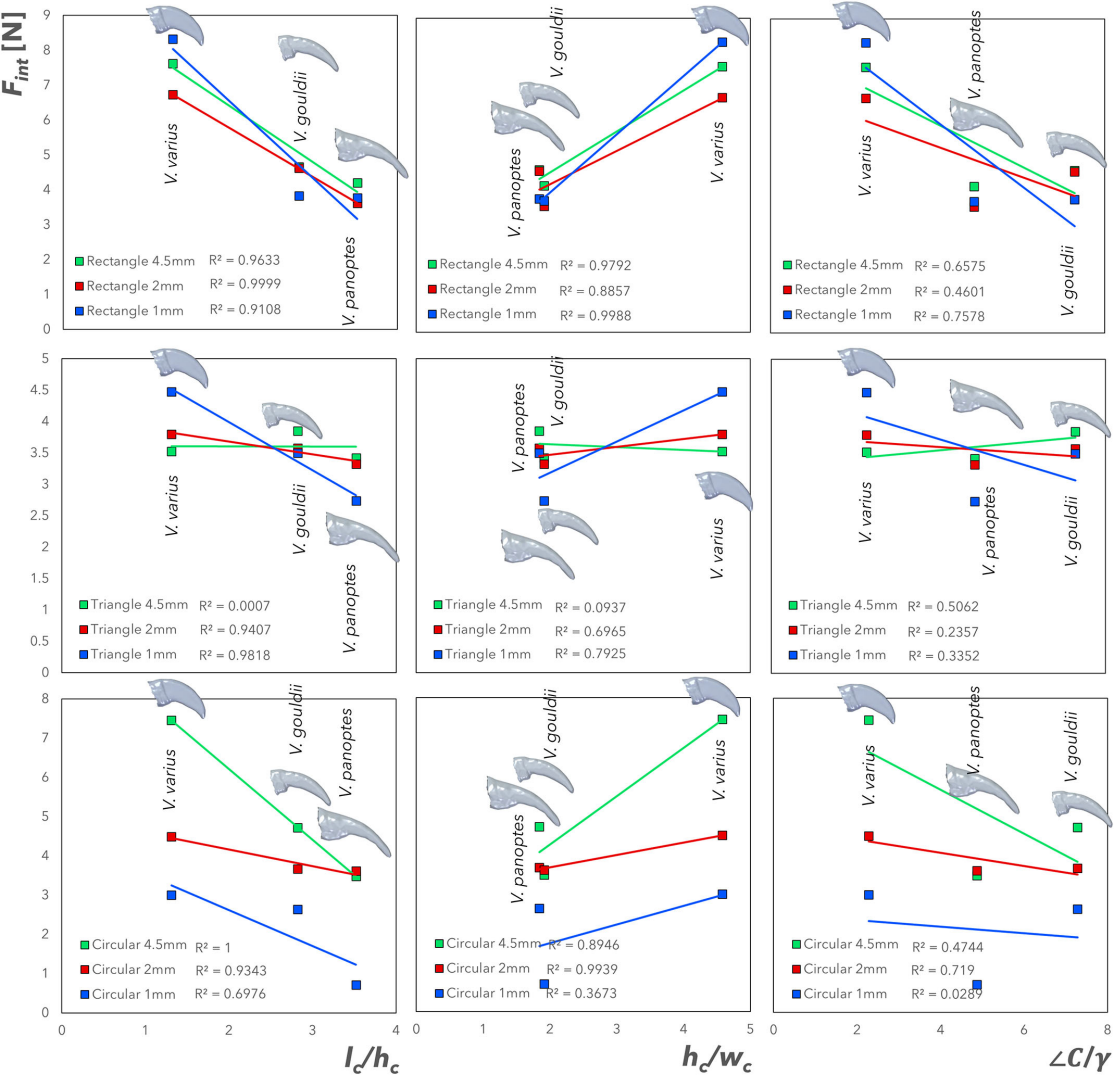
**Mean interlocking grip,** *F*_***int***_**, (*n*=8 for each point) over protuberances (rectangular, triangular and circular at 4.5 mm, 2 mm and 1 mm height) plotted against** 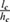
**,** 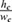
**, and** 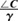 **(cf. Table 2) for each of the lizard species.**

**Fig. 6.**
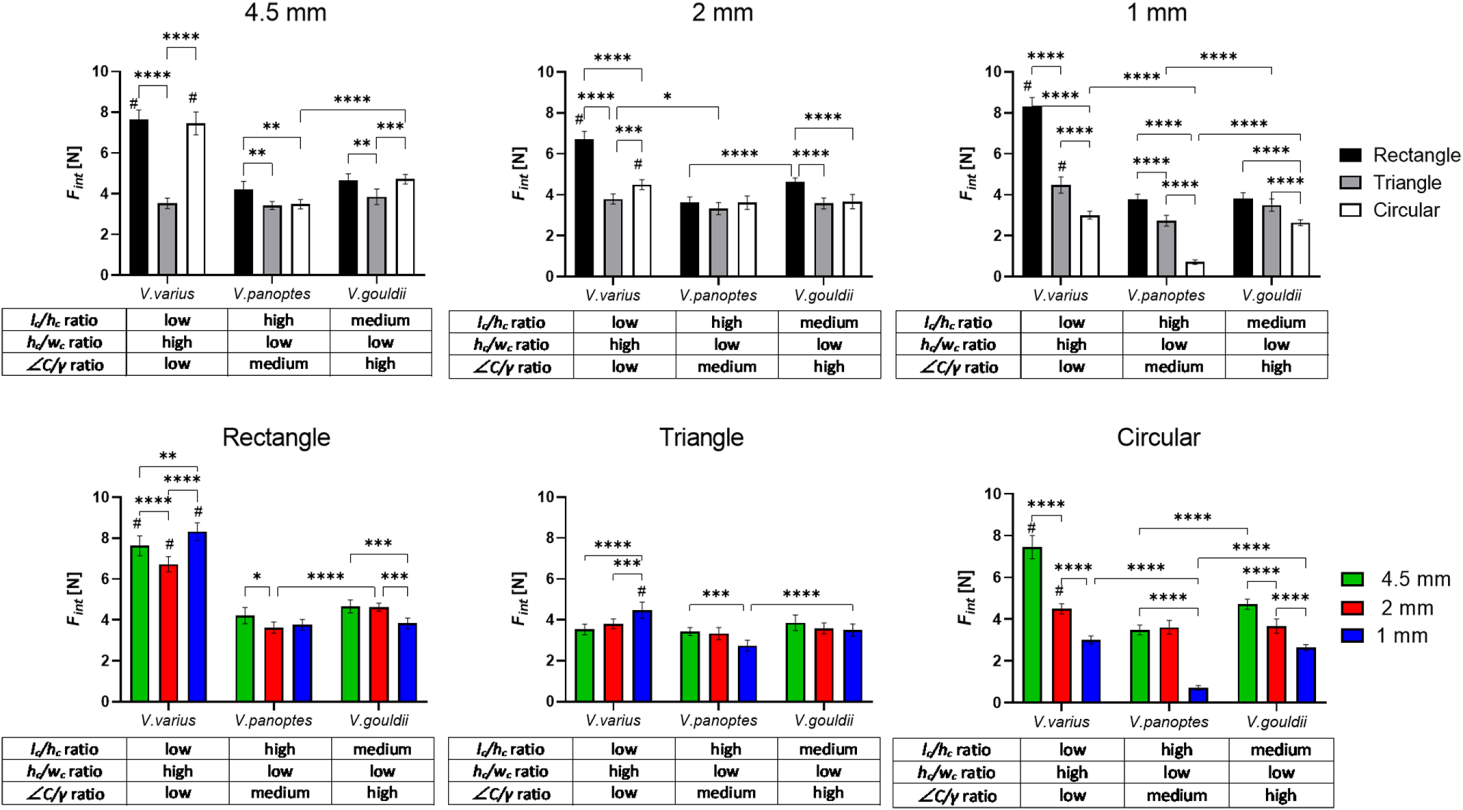
**Comparisons of interlocking force (***F*_***int***_**, species and aspect ratios on different sizes and shapes of surface protuberances, sorted by size of protuberance (upper panel) or shape of protuberance (lower panel).** For simplicity, the aspect ratios for each species are ranked as ‘low’, ‘medium’ or ‘high’ relative to the other two species (see Table 2 for numerical aspect ratio values). The bars indicate mean values ±s.d. Asterisks (*) denote statistically significant differences between mean values of specified groups. ****= *P*<0.0001, ***= *P*<0.001, **= *P*<0.01, *= *P*<0.05, calculated using two-way ANOVA and Tukey's post hoc test. #  = statistically significant difference to both *V. panoptes* and *V. gouldii*, with *P*<0.0001.

Fig. 5 shows the mean interlocking grip, *F*_*int*_, (*n*=8 for each point) over protuberances plotted against 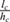, 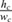, and 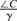 (cf. Table 2) for each of the lizard species. The lateral stability ratio 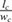 is left out here as it was shown in Fig. 4 to have no discernible relationship with grip force and 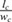 ratios were not significantly different between the three species (cf. Fig. 2). The protuberances are those shown in Fig. 9 (cf. Materials and Methods section) and consist of rectangular, triangular and circular geometries, additively manufactured at heights of 4.5 mm, 2 mm and 1 mm. *F*_*int*_ over rectangular protuberances are at every height, observed as being negatively correlated with the claw aspect 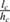, whilst also being positively correlated with the cross-sectional rigidity ratio 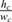. The determination coefficients in each of these cases are high and indicate low levels of scatter from the linear regression line of best fit. This relationship is strongest for the rectangular protuberances, on which the interlocking force of *V. varius* (exhibiting the lowest 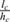 and highest 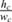) is significantly higher than for the other species, irrespective of protuberance size (Fig. 6). *F*_*int*_ changes differentially on the different protuberance geometries and sizes depending on the claw type, as can be noted from the significant interaction effects presented in the Electronic Table S1. *F*_*int*_ over rectangular protuberances is also negatively correlated with the profile rigidity ratio 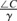; however, there are higher levels of scatter about the best fit line as evidenced by the determination coefficients in this plot. *F*_*int*_ for triangular protuberances, is negatively and positively correlated against 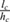 and 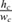, respectively, at heights of 2 mm and 1 mm. However, Fig. 6 shows that *V. varius* only significantly outperforms the other species at the 1 mm height on triangular protuberances. Significant interaction effects (contributing to 33.89% of the total observed variation) can also be noted for the triangular protuberances, (cf. Electronic Table S1 and Fig. 6). At heights of 4.5 mm, there are observably significant inter-species differences and interaction effects in the interlocking force (Fig. 6 and Electronic Table S1) and this is presumably due to a larger alteration of slope angle as a function of protuberance height affecting the interlocking effectiveness of the claws. There is no obvious relationship between *F*_*int*_ and 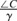. *F*_*int*_ over circular section protuberances is negatively correlated with 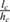, and positively correlated with 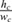, though the scatter about the linear regression best fit increases as the protuberance height decreases. Nevertheless, *V. varius* outperforms the other species on circular protuberances of 4.5 and 2 mm height. Interlocking force also decreases significantly on the 1 mm circular protuberances in all claw types, Fig. 6. Finally, there is no observable correlation between *F*_*int*_ over circular section protuberances and 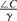 with respect to protuberance height.

The gripping effectiveness on protuberances was measured for each claw type on triangular and circular cross-section protuberances. For ease of comparison and for benchmarking, we define a claw interlocking coefficient, *μ*_*CIC*_, as:
(2)

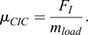
Where the *F*_*I*_ is the mean load that the claw can resist on a protuberance before slipping, and *m*_*load*_ is the constant load applied to the claw normal to the direct of pull. Table 3 shows a choropleth map of the calculated *μ*_*CIC*_ of the three claw types tested on rectangular, triangular and circular cross-section protuberances (*n*=8 for each claw type and sample set). Darker shading indicates a stronger interlocking. Individual *μ*_*CIC*_ values are provided for each test case. *V*. *varius* exhibits an evidently higher concentration of darker shading than is observed for the burrower lizards *V. gouldii* and *V. panoptes*, confirming that the claws of the arboreal lizard *V. varius* exhibit an overall superior capacity for gripping.

**Table 3.**
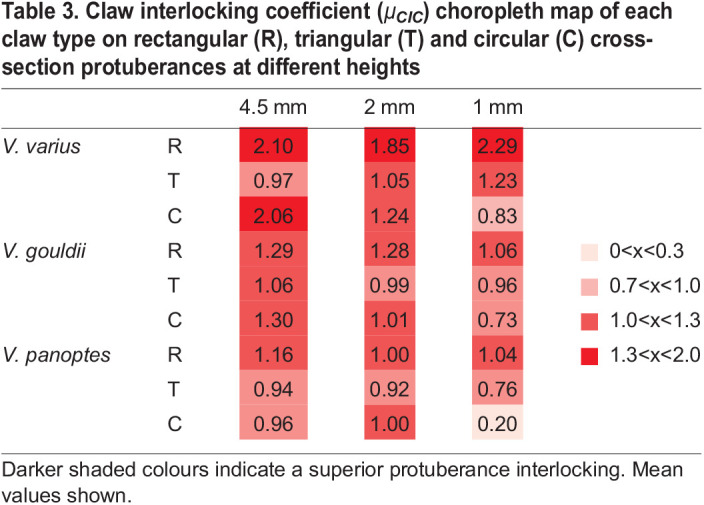
**Claw interlocking coefficient (** *μ*_***CIC***_ **) choropleth map of each claw type on rectangular (R), triangular (T) and circular (C) cross-section protuberances at different heights**

### DISCUSSION

Our morphological measurements show that claws from the arboreal lizard *V. varius* possess a greater claw tip angle and smaller claw curvature, than in the burrower species, *V. gouldii* and *V. panoptes*. Additionally, claws from *V. varius* have the lowest aspect, the highest cross-sectional rigidity ratio, and the lowest profile rigidity ratio out of all the lizard species. These findings bear some resemblance to previous literary reports (Crandell et al., 2014; Zani, 2000) but add additional morphological data and parameters. One morphological aspect not previously covered is claw width. Here, we find that *V. varius* has on average, the thinnest claw, while *V. panoptes*' claw is on average, the widest. This seems consistent with the differences in typical claw use between the arboreal and burrower lizards. Burrowers use their claws to move loose, granular material (e.g. sand) and as such, a wider claw covering a greater surface area may enable the movement of larger volumes of material. Contrarily, when arboreal lizards climb, their claws often interact with substrates by fitting into small irregularities within, and on, the materials. Smaller widths allow the claw to interact with and grip to, a greater range of surface irregularities, fissures or protuberances.

The results of the static grip tests offer several insights when compared against key morphological measurements (c.f. Tables 1 and 2). While individual morphological parameters can be of interest, when comparing between claws of different sizes, normalised morphologies such as those shown in Table 2 are of greater benefit. While there appears to be a generally good relationship between the profile rigidity ratio, 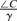, and static grip, *F*_*sg*_ (ca. 26-46% scatter from the regression line), the relationships between *F*_*sg*_ and claw aspect (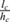) and *F*_*sg*_ and the cross-sectional rigidity ratio (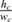) are far more convincing, showing only ca. 0-8% and 1-10% scatter from the regression lines, respectively. The relationships between interlocking grip *F*_*int*_ and 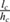, 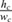 and 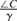 are found to be similar to those of *F*_*sg*_; however, protuberance shape and height have a noticeable effect on the claw interlocking. While rectangular protuberances enable grip at all protuberance heights considered herein, the highest (4.5 mm) triangular protuberances and the lowest (1 mm) circular protuberances do not allow for regularity (and hence repeatability) in gripping, as is evidenced by the high determination coefficients for these sample series. This may be related to the angle at which grip occurs, which is a factor known to affect grip-strength (Lam and Xu, 2011), with angles relative to the direction of pull being generally more effective at <90^°^ (Provancher et al., 2005) alongside coupled parameters such as the sharpness of the claw tip (Pattrick et al., 2018). This concept is important as a lizard has rotating phalangeal joints that essentially control the angle at which a claw can connect to, or penetrate, a substrate. As such, ineffective grip arising from an unfavourable claw angle of attack may well be circumvented in the natural world, by phalangeal joint rotation. When considering individual morphological parameters (cf. Table 1), the smaller curvature 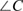, the greater claw tip angle *γ*, smaller claw length *l*_*c*_, and smaller claw width *w*_*c*_, appear to provide *V. varius* a general advantage in terms of both static grip *F*_*sg*_ and interlocking grip *F*_*int*_. Based on comparisons of these grip forces against normalised parameters in Table 2, and illustrated in Figs 4 and 5, it can be claimed that 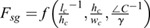; however, the same claim cannot be made for *F*_*int*_, since it does not show a strong correlation with these parameters for all protuberance sizes and shapes. Claw aspect (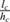) and the cross-sectional rigidity ratio (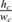) are morphological parameters most prominently influence the ability of a claw to maintain grip with a substrate. Nevertheless, the profile rigidity ratio (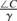) can be considered to be of importance, but not necessarily of equal importance, based on the level of scatter in the 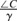 plots versus the scatter observed in the 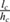 and 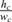 plots of Fig. 5. While other parameters such as substrate roughness (Dai et al., 2002; Ditsche-Kuru et al., 2012) and claw sharpness (Pattrick et al., 2018) have been identified as important to grip strength in insect claws, these parameters have not been taken into account in this study, except in terms of interlocking protuberances and claw tip angle, respectively. When considering the claw interlocking coefficient (*μ*_*CIC*_), we note that in similitude to the *F*_*sg*_ tests, *V. varius* outperforms *V. gouldii* and *V. panoptes*, while *V. gouldii* marginally outperforms *V. panoptes*. The superior interlocking capacity of *V. varius* claws, seems more likely to be a combination of 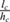, 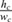 and 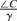, but is not necessarily to linked to any one specific parameter as evidenced by significant interaction effects (cf. Electronic Table S1).

For efficient climbing, a lizard needs to grip to a non-vertical surface (e.g. ledge, fissure) in order to support its weight through its claw. An optimally shaped claw enables efficient climbing since force can be transferred effectively throughout the claw (Mattheck and Reuss, 1991). Here, we find that the arboreal lizard, *V. varius*, has the smallest curvature (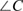), the lowest claw aspect (*A*_*r*_=*l*_*c*_/*h*_*c*_), and the lowest profile rigidity ratio (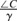), each of which reduces the risk of the claw bending. In particular, the profile-view rigidity ratio 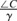 is a curved shape in bending. While ordinarily a rectilinear object in bending resists deformation in both bending and shear, a curved object resists deformation in bending, shear and torsion. A stable, non-deforming contact interface both stabilises the rigidity of the claw on the substrate and may ensure that maximum tractive forces are required to cause slip. Claws with high aspects and higher curvatures are prone to bending stresses (Lautenschlager, 2014), which is both a source of potential damage to the claw and disables its effectiveness as a gripping appendage.

## Conclusion

The claws of the arboreal climbing lizard *V. varius* are morphologically more adapted to climbing than those from the burrower lizards *V. gouldii* and *V. panoptes*. In this paper, we decouple the effects of materials and geometries by 3D printing claws out of the same material, enabling a purely geometrical comparison between the three different claws. We find that there are three key morphological parameters that are important in climbing. These are the claw aspect (a ratio of claw length to claw height), the claw cross-sectional rigidity ratio (a ratio of claw height to claw width) and the profile rigidity ratio (a ratio of claw curvature to claw tip angle). These morphological parameters correlate well with the static grip force; however, with regards to the interlocking grip force, there are coupled size-shape limits that can affect the repeatability of measured interlocking grip forces. In this work, we find that the overall shape of *V. varius* claws are favourable for static gripping and interlocking gripping, and as such the claws of this lizard are deemed geometrically superior for climbing when compared to those of *V. gouldii* and *V. panoptes*.

## MATERIALS AND METHODS

### Claw procurement

Images and claws were taken from the following lizards, *V. varius, V. gouldii* and *V. panoptes*. The claws were dried and cleaned in 70% ethanol. Lizards were collected under permits WISP11435612 (QLD), SF009075 (WA), 61540 (NT), 08-001092-5 (WA) and WA0001919 (QLD); and ethics SBS/195/12/ARC (QLD), ANA16104 (QLD) and RA/3/100/1188 (WA). The age of animals is not reported as they were largely caught from the wild, with the vast majority of the animals caught being males.

### 3D-scanning and 3D printing of claws

**Fig. 7.**
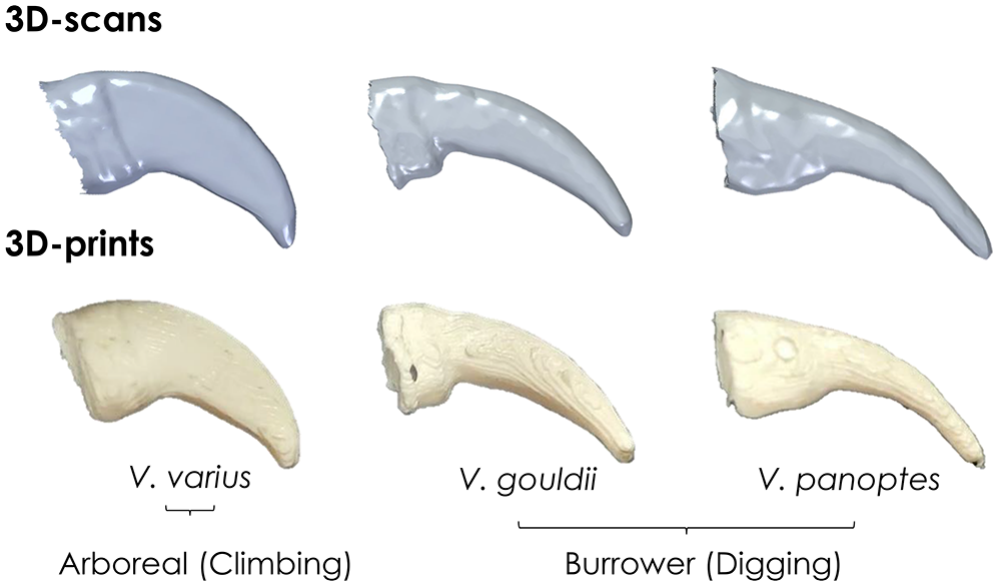
3D-scans and 3D-prints of claws from *V*. *varius*, *V*. *gouldii* and *V*. *panoptes*.

Claws cut at the distal phalanx from the fourth digit of the front right foot from two dead adult lizards from each species were scanned using an Einscan Pro 2X Plus, which can scan to an accuracy of 40 µm in fixed-scan mode. The scanner was mounted and used in conjunction with a fixed turntable, upon which the claw samples were affixed. The scanner uses visible white light and processes up to 1500000 points per second at 30 fps. Individual scans were incremented at 45° within turntable coded targets, each individual scan pieced together automatically by the Einscan software (Version 3.403). A watertight mesh was used to output scanned information in stereolithographic (.stl) format. An Ultimaker 3 fused deposition modelling (FDM) 3D printer was used to additively manufacture claws for testing (0.4 mm nozzle diameter). To ensure that materials properties would not be an independent variable in our tests, we manufactured all claw replicates using poly-lactic acid (PLA). The PLA used in this study has an elastic modulus of 3.071 GPa, which is slightly higher than the *β*-keratin sheaths covering ostrich claws (1.84 GPa) but similar to the *β*-keratin of horse hoof keratin (2-3 GPa) (Bonser, 2000). We surface finished the claws by sanding to remove extraneous material, until the claws were geometrical replicates of the original claws and their scans. Fig. 7 shows both the 3D scans and final 3D prints of the claws from each species or interest. The utility of artificial claws as a tool to evaluating claw performance has previously been carried for dinosaur claws by Manning et al. (2006), who used a robotic limb in conjunction with claws manufactured using an aluminium core and sheaths of Kevlar and carbon fibre strands.

### Morphological measurements

**Fig. 8.**
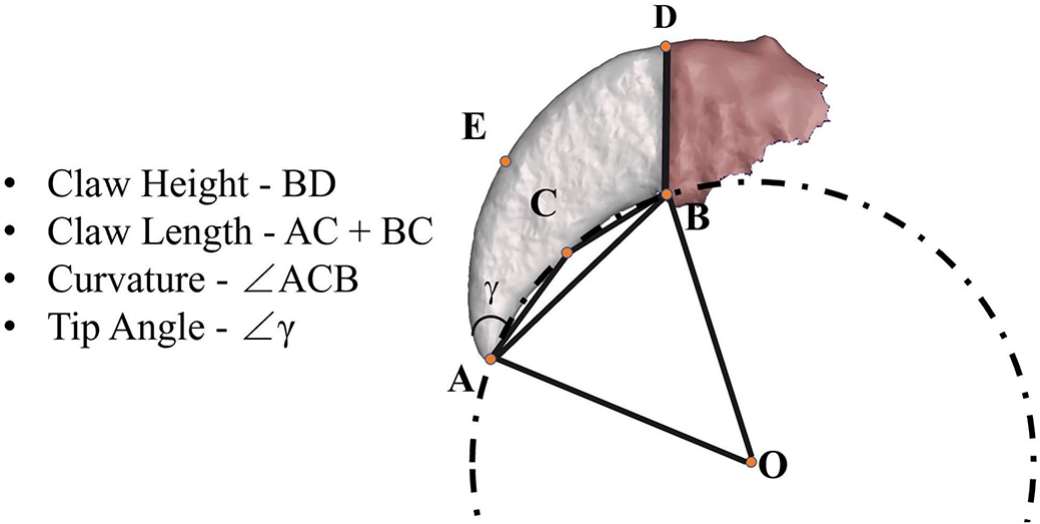
Morphological measurements.

3D scans were initially used to map key morphological features on each claw. The 3D scan of *V. varius* is used in Fig. 8 to illustrate the parameters used to make our measurements. Point A is at the point of the claw tip. Points B and D are defined as the bottom and the top points of the claw base, respectively, where the claw meets the claw sheath (claw sheath shown in red). Point C, referred to herein as the ‘inner reference point’, is defined as either the inflection point of the innerclaw (point at which there is a change in curvature) or if there are multiple or no inflection points, the point at which the perpendicular bisector of line AB intersects with the inner curvature of the claw. Similarly, Point E, the ‘outer reference point’ is defined as either the point at which the perpendicular bisector of AB intersects with the outer curve of the claw, or the inflection point of the outer claw. The dotted line shown in Fig. 8 is a part of a circle drawn through points A, B and C with point O being the centre of the circle. This circle helps illustrate how angle ACB approximately reflects the curvature (degree of curving) of the claw. The claw height, *h*_*c*_, is the distance BD, where the base of the claw emerges from the claw sheath and the measurement is taken orthogonally at this point. The claw length, *l*_*c*_, is defined as the sum of lengths AC and CB. The claw width, *w*_*c*_, is defined as the thickness of the claw at point C. The curvature, 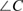, is defined as angle ACB. It is important to note that a gentle curve is likely to have a greater curvature angle, while a smaller curvature will correspond to a tighter curve. To calculate the curvature therefore, we use the cosine theorem, Eqn 1. Finally, to define the tip angle, *γ*, we drew an additional circle using Points A, E and D. This results in two circles, one including Points A, C, and B and the other including points A, E, and D, which intersect at Point A. The tangents of these two circles are drawn at Point A and the angle between these tangents is defined as the tip angle. Here, a smaller tip angle correlates with a sharper claw tip. Based on the parameterisation conducted on the 3D scans, images of claws from individual digits of each lizard species were morphologically analysed. A total of 27 measurements were made for *V. varius*, 14 for *V. gouldii* and 10 for *V. panoptes*.
(1)

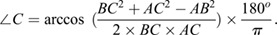


### Static grip and interlocking

**Fig. 9.**
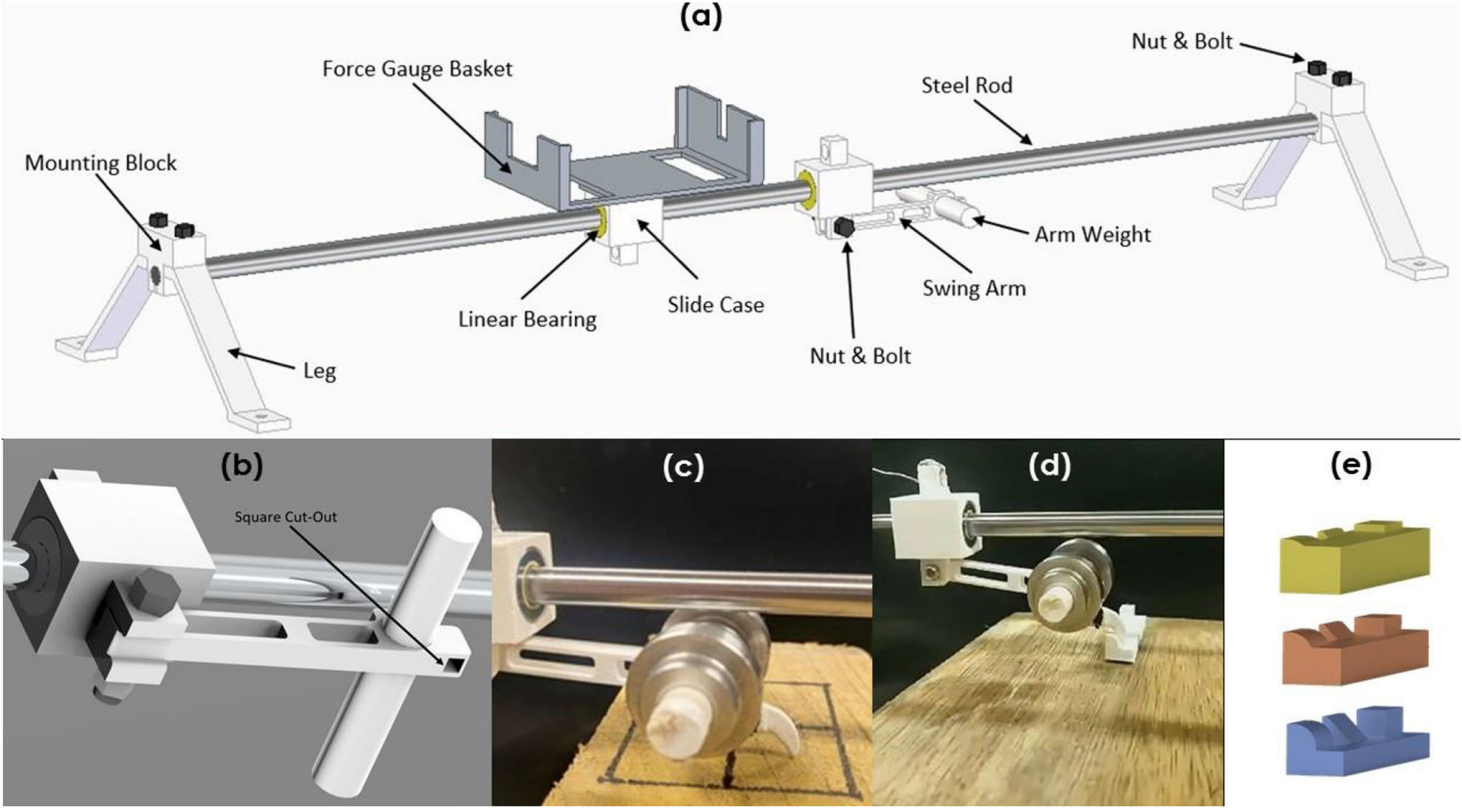
**(A) CISPA with its parts labelled.** (B) cuboidal slot (CISPA arm) into which a 3D printed claw is inserted. (C) 3D printed claw inserted into CISPA and resting on surface ready for measurement of static grip, *F*_*sg*_. (D) Interlocking test set up with interlocking protuberances affixed to a wooden table and (E) 3D printed protuberances for interlocking experiments at heights of (top to bottom) 1 mm, 2 mm and 4.5 mm.

There are currently no purpose-built devices that enable the testing of a claw's performance in gripping or interlocking. As such we designed a Claw Isolating Static Pulling Apparatus (CISPA) to quantitatively determine the forces required to displace a claw resting on a surface or over an obstacle. The fundamental set-up of CISPA is shown in Fig. 9A. CISPA has been designed to (a) isolate the gripping action of the claw as a function of geometry and (b) enable claw testing on a range of different surfaces and protuberances. With CISPA, using an electronic scale that measures force in tension (Newton measurements converted to weight measurements), we are able to measure force at the moment the claw slips from its static state (static grip, *F*_*sg*_) when a constant horizontal pull is applied to it, resulting in an increasing force. Claw samples are 3D printed with a cuboidal insert attached. The insert fits into CISPA on the underside of the arm (c.f. Fig. 9A) in front of the arm weight (Fig. 9B and C).

Initial static grip tests were carried out on wood, stone and polished ice substrates. Each claw from each species was tested eight times on each substrate, totalling 72 tests. Tests were conducted on different areas of each substrate to avoid testing bias. Prior to conducting the tests, we determined the static coefficient of friction of PLA on each of the substrates. The ratio of the static friction coefficient *μ* of PLA on wood, stone and ice was found to be *μ*_*w*_:*μ*_*b*_:*μ*_*i*_=1:1.28:0.05, in respective order.

Interlock tests were then conducted using CISPA to measure the maximum force required for each 3D printed claw replicate to break interlock staticity. Rectangular, triangular and circular cross-sectioned interlocking protuberances were 3D printed in PLA. The gripping performance of each of the claw replicates was tested against these protuberances (Fig. 9D). To additionally assess the effects of protuberance size on interlocking, each of the shapes was manufactured at heights of 1 mm, 2 mm, and 4.5 mm (Fig. 9E). The height here is defined as the vertical distance between the top point of the shape and the flat bottom surface (base) that the protuberances extend from. Videos were taken at 60 fps to deduce the point at which claw interlocking staticity was broken and the force recorded at this slip point. Each claw replicate was tested eight times on each protuberance type, and at each protuberance height. A video example of a test over a protuberance is provided as Electronic Supplementary Movie 1.

### Statistical analyses

All statistical analyses were performed using the GraphPad Prism V9 software. One-way ANOVA and Tukey's post hoc test were used to test statistical significance (*P*<0.05) between the mean values of the aspect ratios for different claw types. Two-way ANOVA and Tukey's post hoc test were used for statistical significance testing of (a) differences in the static grip forces between intra- and inter-species' claw types over different flat substrates and (b) differences in the interlocking forces between intra- and inter-species' claw types over different protuberance heights and shapes, as well as for analysing their interaction effects.

## Supplementary Material

10.1242/biolopen.059874_sup1 Supplementary information
