# Supplementary material for: The influence of claw morphology on gripping efficiency
Source: Biol Open. 2023 May 16;12(5):bio059874. doi: 10.1242/bio.059874 (PMC10214847; doi:10.1242/bio.059874)
Supplement: Supplementary information [file biolopen-12-059874-s1.pdf]

Table S1. Interaction effects between protuberance geometries and claw type as well as size of protuberance and claw type. The percentages express the % of total variation that originates from the interaction effects calculated using Two-Way ANOVA.

Variable		Interaction effect, % of total variation	p-value
Protuberance size	4.5 mm	23.50	<0.0001
	2 mm	22.16	<0.0001
	1 mm	15.74	<0.0001
Protuberance geometries	Rectangle	5.62	<0.0001
	Triangle	33.89	<0.0001
	Circular	11.98	<0.0001

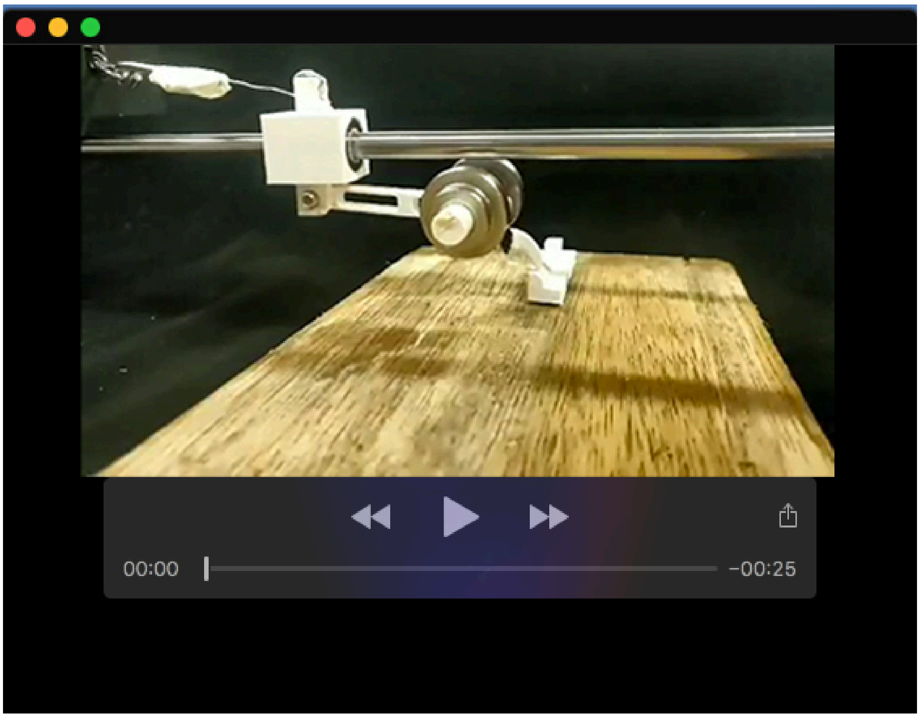

Movie 1.

Biology Open • Supplementary information
